# Effects of nasal strip on efficiency of non-invasive ventilation in patients with acute respiratory failure, preliminary results

**DOI:** 10.1186/2197-425X-3-S1-A676

**Published:** 2015-10-01

**Authors:** DM Yavşan, R Altuntaş, H Caner, S Demirbaş, M Göktepe, T Teke, K Uzun

**Affiliations:** Necmettin Erbakan University Meram Medical Faculty, Pulmonary Diseases and Critical Care Unit, Konya, Turkey

## Objectives

Noninvasive ventilation (NIV) is considered the standart of care in the management of acute hypercapnic respiratory failure due to COPD. NIV failure has been defined as the need for endotracheal intubation or death. Its rate greatly varies between 5-60%, depending on numerous factors. The choice of interface during NIV represents the main determinant of its success in an acute setting. Asynchrony has rarely been cited as a direct cause of NIV immediate failure. We aimed effect of nasal strips in success of NIV in acute hypercapnic respiratory failure.

## Methods

This study was conducted in a 12-bed adult respiratory intensive care unit (RICU) (>18 years). We evaluated 53 COPD patients with acute hypercapnic respiratory failure. Patients divided to two groups (Nasal strip; NIV with nasal strip (n=29), Control; NIV without nasal strip (n=24). Median age of groups were 66.93 ± 13.8 years (control) and 68.33 ± 11.7 years (group nasal strip). The median APACHE II score on admission were 14.16 ± 5.98 (group nasal strip) and 14.58 ± 5.38 (control).

## Results

The results of study was shown in Table 1. There were differences according SAPS II score, tidal volume (VT) and need of FiO_2_ between nasal strip group and control group (p < 0.05). There were no association with mortality and length of stay in RICU.

**Table 1:** The results in groups according to using nasal str.

	APACHE II Score	SAPS II Score	Length of Stay (day)	Tidal Volume (mL)	Respiratory Rate (/minute)	FIO2	pH	PaCO2 (mmHg)	PaO2 (mmHg)
Nasal Strip Group	12.8 ± 8.4	27.07 ± 10.3	9.3 ± 5.8	524.8 ± 178.5	12 ± 0.6	0.41 ± 0.04	7.32 ± 0.06	54.5 ± 15.4	90.1 ± 19.6
Control Group	14.2 ± 12.9	66.2 ± 34.8	13.1 ± 11.6	441.5 ± 96.45	12.1 ± 0.5	0.45 ± 0.08	7.32 ± 0.09	53.9 ± 17.6	83.7 ± 19.3
P value	>0.05	< 0.05	>0.05	< 0.05	>0.05	< 0.05	>0.05	>0.05	>0.05

## Conclusions

Nasal strips may helpful and improve the NIV success in the icu patients suffer from acute respiratory failure with COPD.

